# Iron Deprivation by Oral Deferoxamine Application Alleviates Acute Campylobacteriosis in a Clinical Murine *Campylobacter jejuni* Infection Model

**DOI:** 10.3390/biom13010071

**Published:** 2022-12-29

**Authors:** Stefan Bereswill, Soraya Mousavi, Dennis Weschka, Agnes Buczkowski, Sebastian Schmidt, Markus M. Heimesaat

**Affiliations:** 1 Gastrointestinal Microbiology Research Group, Institute of Microbiology, Infectious Diseases and Immunology, Charité—Universitätsmedizin Berlin, Corporate Member of Freie Universität Berlin, Humboldt-Universität zu Berlin, and Berlin Institute of Health, 12203 Berlin, Germany; 2 Hofmann & Sommer GmbH und Co. KG, Büro Berlin, 12489 Berlin, Germany

**Keywords:** desferoxamine, iron binding, enteropathogenic infection, *Campylobacter jejuni*, immune-modulatory effects, disease-modifying properties, microbiota-depleted IL-10^−/−^ mice, acute campylobacteriosis model, host-pathogen interaction, preclinical intervention study, natural antibiotics-independent compounds, reactive oxygen species

## Abstract

The progressively rising food-borne *Campylobacter jejuni* infections pose serious health problems and socioeconomic burdens. Given that antibiotic therapy is not recommended for most campylobacteriosis patients, novel treatment options include strategies targeting iron homeostasis that impacts both *C. jejuni* virulence and inflammatory cell damage caused by toxic oxygen species. In our preclinical intervention study, we tested potential disease-alleviating effects upon prophylactic oral application of the iron-chelating compound desferoxamine (DESF) in acute murine campylobacteriosis. Therefore, microbiota-depleted IL-10^−/−^ mice received synthetic DESF via the drinking water starting seven days before oral infection with *C. jejuni* strain 81-176. Results revealed that the DESF application did not reduce gastrointestinal pathogen loads but significantly improved the clinical outcome of infected mice at day 6 post-infection. This was accompanied by less pronounced colonic epithelial cell apoptosis, attenuated accumulation of neutrophils in the infected large intestines and abolished intestinal IFN-γ and even systemic MCP-1 secretion. In conclusion, our study highlights the applied murine campylobacteriosis model as suitable for investigating the role of iron in *C. jejuni* infection in vivo as demonstrated by the disease-alleviating effects of specific iron binding by oral DESF application in acute *C. jejuni* induced enterocolitis.

## 1. Introduction

Campylobacteriosis constitutes a frequent infectious enteritis syndrome that is caused by Gram-negative bacterial enteropathogens of the genus *Campylobacter* [1]. In the majority of reported cases, food-borne *C. jejuni* represent the infectious agents that are transferred from non-symptomatic livestock including poultry to humans by ingestion of contaminated and undercooked meat products [2]. In humans, the pathogens cause clinical manifestations of infectious enteritis such as abdominal pain, vomiting, headache, fever, and diarrhea, sometimes with bloody stools [3]. The rising prevalence of *C. jejuni* infections worldwide represents a significant burden to the global health system [3,4,5]. In the majority of cases *C. jejuni* infections are self-limiting and resolve without residue within 14 days. Infected patients with mild symptoms usually receive symptomatic treatment such as fluid and electrolyte replacement and analgesic compounds. Antibiotic treatment, however, is in place for severely diseased patients who are at high risk for systemic complications [5]. The ability of *C. jejuni* to cause disease depends on the production of bacterial pathogenicity factors [6]. Motility, adhesion, and tissue invasiveness of *C. jejuni* play major roles in the onset of intestinal inflammation that is triggered by the bacterial endotoxin lipo-oligosaccharide (LOS). Within weeks to months after the initial infection, rare but severe post-infectious sequelae such as Guillain–Barré syndrome, irritable bowel syndrome, celiac disease, inflammatory bowel diseases, and reactive arthritis might manifest [1,7].

The pathogenesis of human campylobacteriosis is dominated by innate immune responses that are triggered and aggravated by the LOS of the invading *C. jejuni* bacteria. In consequence, the histopathology of intestinal biopsies taken from campylobacteriosis patients is characterized by infiltrates of innate immune cells such as neutrophilic granulocytes, macrophages, monocytes, and lymphocytes as well as by increased numbers of apoptotic epithelial cells representing hallmarks of the histopathological diagnosis of campylobacteriosis [8]. The immune cells produce reactive oxygen species (ROS) and pro-inflammatory mediators, which cause apoptosis of epithelial cells [9]. Cell death results in epithelial barrier destruction, malabsorption, and diarrhea [10]. Thus, in most cases campylobacteriosis presents as a leaky gut syndrome. However, more severe and even systemic manifestations may occur in immunocompromised individuals [3,11]. Both severity of enteritis as well as the risk for post-infectious morbidities are significantly associated with the molecular LOS structure of the infecting *C. jejuni* strain, further underlining the central role of LOS in the pathogenesis of campylobacteriosis [12].

Within a week after oral *C. jejuni* infection, microbiota-depleted IL-10^−/−^ mice display LOS-driven human campylobacteriosis [10,13,14]. In these infected animals, bloody diarrhea caused by epithelial apoptosis and inflammatory immune responses is initiated by signaling of the *C. jejuni*-LOS via Toll-like receptor-4 [14,15]. Immune cells infiltrating infected intestinal sites induce enteric, but also extra-intestinal and even systemic inflammatory responses in severe disease [16]. This murine campylobacteriosis model was successfully applied for the preclinical validation of novel therapy options for campylobacteriosis including sirolimus [13]; vitamin C [17]; vitamin D [18]; carvacrol [19]; resveratrol [20,21]; urolithin-A [22]; essential oils derived from cardamom, clove, cumin, and garlic [23,24,25,26]; neuropeptides [27,28]; probiotics [29]; fecal microbiota transplantation [30,31]; and activated charcoal [32].

To further extend novel treatment options for human campylobacteriosis we investigated the iron chelator deferoxamine B (desferrioxamine, DESF; trade name Desferal^®^), which combines antibiotic, anti-inflammatory, and cell protective properties in one molecule [33]. In its biological role as a natural siderophore produced by microorganisms such as *Streptomyces pilosus* to obtain iron from the environment, the main mode of action of DESF is the binding of iron. Thereby, DESF exerts effects which may protect the human body from iron intoxication and enteropathogenic invasion [34]. In consequence, DESF is effectively used for treatment of iron storage diseases such as hemochromatosis or thalassemia major in which excess iron within cells drives DNA destruction and cell death by ROS production via the Fenton reaction [34]. Moreover, given that *C. jejuni* and many other pathogens depend on iron for replication and energy supply, free iron is effectively bound to transferrin, lactoferrin, and ferritin, and furthermore, forms part of hemoglobin in healthy individuals. Thus, iron binding by DESF supports this function and reduces the risk for infections [34]. The application of DESF as a treatment for bacterial and parasitic infections has been proposed for decades [35], and iron-scavenging might ameliorate viral hyper-ferritinemic diseases including COVID-19 [36].

*C. jejuni* is not able to produce siderophores, and in contrast to enterobactin-bound iron, it cannot use DESF-bound iron as an iron-delivering agent. The pathogen imports ferrous iron directly and can use siderophores produced by other microorganisms such as enterobactin as well as the host molecules heme, lactoferrin and transferrin as iron sources [37,38,39,40]. In the absence of these iron sources in vitro, DESF in concentrations in the range of 10 mg/L (≈20 µM) inhibits *C. jejuni* growth completely. This antimicrobial activity directed against *C. jejuni* is similar to that of ciprofloxacin used for the treatment of campylobacteriosis in immunocompromised patients [40]. In addition, DESF treatment might interrupt environmental adaptation and virulence of *C. jejuni*. Even flagellar synthesis that is essential for motility of the pathogen and required for disease induction is activated by iron [41].

Beneficial effects of DESF in the treatment of intestinal inflammation are further supported by studies showing effective treatment of colitis in rats with DESF [42,43] and by results from a clinical study demonstrating disease-alleviating effects of DESF in intestinal biopsies taken from ulcerative colitis patients [44].

Given the multifaceted health-beneficial features of DESF, including antimicrobial, anti-inflammatory, cell protective, and regulatory, we studied its possible disease-alleviating effects in acute campylobacteriosis by using *C. jejuni* infected microbiota-depleted IL-10^−/−^ mice as a clinical infection model. Given that DESF is poorly absorbed in the intestinal tract [45], we used oral application of the compound in order to test for pathogen clearance in line with dampening of intestinal inflammation, both required for amelioration of campylobacteriosis. We therefore surveyed gastrointestinal pathogen loads, clinical outcome, colonic histopathology, and epithelial cell apoptosis in line with intestinal and systemic immune responses upon *C. jejuni* infection of microbiota-depleted IL-10^−/−^ mice that had been pretreated with DESF via the drinking water starting a week prior infection in order to drain intestinal iron reservoirs.

## 2. Materials and Methods

### 2.1. Microbiota-Depleted IL-10^−/−^ Mice

C57BL/6j IL-10^−/−^ mice were obtained from the Forschungsinstitute für Experimentelle Medizin, Charité—Universitätsmedizin Berlin, Germany and maintained under standard conditions as reported earlier [46]. The commensal gut microbiota was depleted by an 8-week treatment with ampicillin plus sulbactam (2 g/L; Dr. Friedrich Eberth Arzneimittel, Ursensollen, Germany) via the autoclaved drinking water (ad libitum) starting immediately after weaning (i.e., at the age of 3 weeks) as described recently [46]. Immediately before starting the DESF pretreatment (i.e., 7 days prior infection), the antibiotic solution was replaced by autoclaved tap water supplemented with DESF (see below). Microbiota-depleted animals were kept and handled under strict aseptic conditions.

### 2.2. Campylobacter jejuni Infection and Desferoxamine Pretreatment

The *C. jejuni* strain 81-176 used for the experiments was grown on selective karmali agar plates (from Oxoid, Wesel, Germany). Age- and sex-matched microbiota-depleted IL-10^−/−^ mice (3-month-old littermates) were infected perorally with 10^9^ colony forming units (CFU) of the pathogen on days 0 and 1 by gavage. Mice were treated with DESF (purchased from Sigma-Aldrich, Munich, Germany) added to the autoclaved drinking water (final concentration of 500 mg/L; ad libitum) starting 7 days prior infection. This DESF concentration was chosen to exceed the iron concentration of ≈160 mg/kg in the mice feeds (standard food pellets: sniff R/M-H, V1534-300; Sniff, Soest, Germany; ad libitum). Mice from the placebo (PLC) cohort received autoclaved tap water only.

### 2.3. Gastrointestinal Pathogen Concentrations

After *C. jejuni* infection, the pathogen loads (with a detection limit of 100 bacteria per g) of intraluminal gastrointestinal samples and feces were determined by a culture of serial dilutions plated onto karmali agar plates (Oxoid, Wesel, Germany) as described previously [15].

### 2.4. Clinical Aspects

The daily clinical conditions of mice were surveyed and quantified by using a cumulative clinical score (maximum 12 points) as described earlier [47].

### 2.5. Sampling Procedures and Measurement of Inflammatory Mediators

Mice were sacrificed by CO_2_ asphyxiation on day 6 p.i. and blood was obtained by cardiac puncture. Colonic samples were collected from each mouse for subsequent microbiological and immunohistopathological analyses and ex vivo biopsies from the liver, kidneys, lungs, mesenteric lymph nodes (MLN), ileum and colon as well as luminal samples from the stomach, duodenum, ileum, and colon were derived under aseptic conditions. The serum samples and ex vivo biopsies were used for the determination of inflammatory mediators by the Mouse Inflammation Cytometric Bead Assay (CBA; BD Biosciences, Heidelberg, Germany) on a BD FACSCanto II flow cytometer (BD Biosciences, Heidelberg, Germany) as described earlier [15].

### 2.6. Histopathological Analysis and Immunohistochemistry

Paraffin-embedded colonic ex vivo biopsies were used for histopathological analyses by light microscopy (100 × magnification) and histopathological changes in the large intestines were quantified by scoring as described earlier [48]. Quantitative in situ immunohistochemical analyses of apoptotic epithelial cells, macrophages and monocytes, neutrophilic granulocytes, T lymphocytes, regulatory T cells, and B lymphocytes, colonic paraffin sections (5 µm) were performed as described [49]. After specific staining, cells were quantitated by a blinded independent investigation using light microscopy. The average number of respective positively stained cells in each sample was determined within at least six high power fields (HPF, 0.287 mm^2^, 400 × magnification).

### 2.7. Statistics

Medians and significance levels were calculated using GraphPad Prism (version 9; San Diego, CA, USA). Normalization of data was assessed by the Anderson–Darling test. The Student’s t test and the Mann–Whitney test were applied for pairwise comparisons of normally and not normally distributed data, respectively. For multiple comparisons, the one-way ANOVA with Tukey post-correction (for normally distributed data) and the Kruskal–Wallis test with Dunn’s post-correction (for not normally distributed data) were performed. Two-sided probability (*p*) values ≤ 0.05 were considered significant. Data were pooled from three independent experiments. Definite outliers were identified by the Grubb’s test (α = 0.001).

## 3. Results

### 3.1. Pathogenic Loads in the Gastrointestinal Tract Following C. jejuni Infection of Microbiota-Depleted IL-10^−/−^ Mice That Had Been Pretreated with Deferoxamine

**Figure 1:**
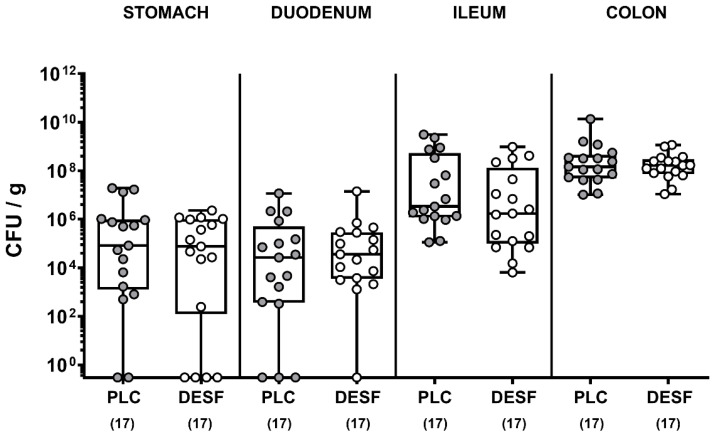
Pathogenic loads in the gastrointestinal tract following *C. jejuni* infection of microbiota-depleted IL-10^−/−^ mice that had been pretreated with deferoxamine. Microbiota-depleted IL-10^−/−^ mice were subjected to desferoxamine (DESF, white circles) pretreatment via the drinking water starting 7 days prior infection or received autoclaved tap water only (PLC; grey circles). On days 0 and 1, mice were perorally infected with *C. jejuni* strain 81-176 and the luminal pathogen loads (expressed as colony-forming units per gram; CFU/g) were quantitatively assessed in defined parts of the gastrointestinal tract by culture on day 6 post-infection. Whiskers indicating the total range, boxes indicating the 25th and 75th percentiles of the medians (black bars inside boxes), and the total numbers of analyzed mice (in parentheses) are given. Shown data were pooled from three independent experimental sets.

We first assessed whether oral DESF pretreatment of *C. jejuni* infected microbiota-depleted IL-10^−/−^ mice resulted in lower pathogen numbers in distinct gastrointestinal compartments. Our cultural analyses revealed, however, that *C. jejuni* cell counts did not differ in luminal samples taken from the stomach, duodenum, ileum, and colon of DESF as compared to PLC pretreated mice on day 6 p.i. (*p* > 0.05; not significant (n.s.), Figure 1). Hence, oral DESF pretreatment did not exert antibacterial effects against *C. jejuni* in the gastrointestinal tract of infected mice.

### 3.2. Kinetic Survey of Clinical Conditions Following C. jejuni Infection of Microbiota-Depleted IL-10^−/−^ Mice That Had Been Pretreated with Deferoxamine

**Figure 2:**
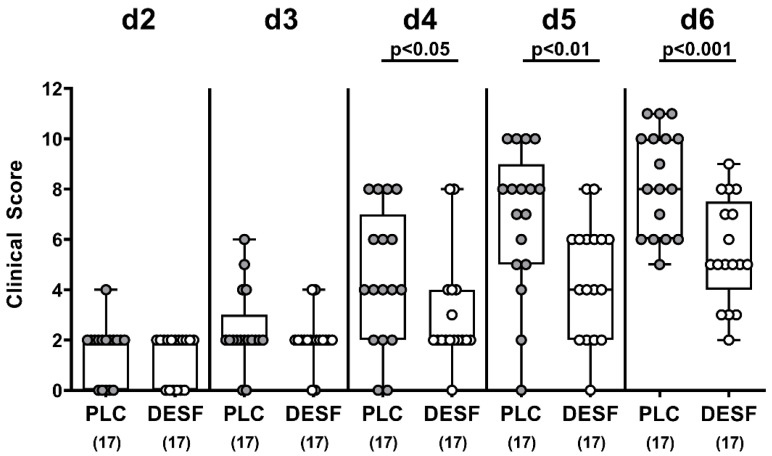
Kinetic survey of clinical conditions following *C. jejuni* infection of microbiota-depleted IL-10^−/−^ mice that had been pretreated with deferoxamine. Microbiota-depleted IL-10^−/−^ mice were subjected to desferoxamine (DESF, white circles) pretreatment via the drinking water starting 7 days prior infection or received autoclaved tap water only (PLC; grey circles). On days 0 and 1, mice were perorally infected with *C. jejuni* strain 81-176. The clinical conditions of mice were quantitatively surveyed over time by a clinical scoring system assessing stool consistency, abundance of fecal blood and wasting symptoms (refer to methods). Whiskers indicating the total range, boxes indicating the 25th and 75th percentiles of the medians (black bars inside boxes), significance levels (*p* values) calculated by the Mann–Whitney U test, and the total numbers of analyzed mice (in parentheses) are given. Shown data were pooled from three independent experimental sets.

We further quantitatively surveyed the clinical signs of *C. jejuni* infection upon DESF pretreatment. In fact, as early as 4 days p.i., DESF pretreated mice were less distinctly suffering from *C. jejuni* induced disease when compared to PLC counterparts as indicated by lower clinical scores in the former versus the latter (*p* < 0.05), which also held true for days 5 and 6 p.i. (*p* < 0.01 and *p* < 0.001, respectively; Figure 2). Hence, oral DESF pretreatment resulted in an improved clinical outcome in *C. jejuni* infected mice.

### 3.3. Microscopic Inflammatory Responses in the Colon Following C. jejuni Infection of Microbiota-Depleted IL-10^−/−^ Mice That Had Been Pretreated with Deferoxamine

**Figure 3:**
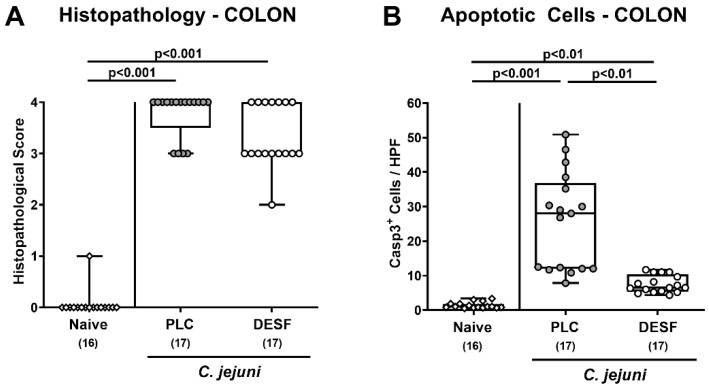
Microscopic inflammatory responses in the colon following *C. jejuni* infection of microbiota-depleted IL-10^−/−^ mice that had been pretreated with deferoxamine. Microbiota-depleted IL-10^−/−^ mice were subjected to desferoxamine (DESF, white circles) pretreatment via the drinking water starting 7 days prior infection or received autoclaved tap water only (PLC; grey circles). On days 0 and 1, mice were perorally infected with *C. jejuni* strain 81-176. On day 6 post-infection, microscopic inflammatory responses were assessed in colonic paraffin sections by quantification of (**A**) histopathological changes applying a standardized histopathological score and of (**B**) apoptotic colonic epithelial cells following immunohistochemical staining with an antibody against cleaved caspase-3 (Casp3). Indicated are the medians of Casp3^+^ cells within six representative high-power fields (HPF, 400× magnification). Uninfected and untreated mice served as negative controls (white rhombus). Whiskers indicating the total range, boxes indicating the 25th and 75th percentiles of the medians (black bars inside boxes), significance levels (*p* values) calculated by the Kruskal–Wallis test with Dunn’s post-correction, and the total numbers of analyzed mice (in parentheses) are given. Shown data were pooled from three independent experimental sets.

We then addressed whether the improved clinical outcome upon DESF pretreatment was associated with alleviated microscopic inflammatory sequelae of *C. jejuni* infection. To test this, we quantitated inflammatory changes in the colonic mucosa on day 6 p.i. by histopathological scores. Whereas PLC treated mice were suffering from severe enterocolitis, there was a trend towards lower histopathological scores in DESF treated mice with median values indicative for moderate inflammatory changes in the colonic mucosa (*p* > 0.05, n.s.; Figure 3A).

Since apoptosis is regarded as a suitable marker for the grading of inflammatory conditions in the intestinal tract including *C. jejuni* induced enteric morbidities [15], we further expanded our microscopic inflammatory analyses for in situ immunohistochemical staining of colonic paraffin sections with an antibody directed against cleaved caspase-3. *C. jejuni* infection resulted in increased numbers of caspase-3^+^ colonic epithelial cells within 6 days (*p* < 0.01–0.001; Figure 3B), whereas this increase was, however, less pronounced in DESF as compared to PLC treated mice (*p* < 0.001; Figure 3B). Hence, DESF pretreatment dampened *C. jejuni* induced apoptotic cell responses in colonic epithelia.

### 3.4. Colonic Immune Cells Responses Following C. jejuni Infection of Microbiota-Depleted IL-10^−/−^ Mice That Had Been Pretreated with Deferoxamine

**Figure 4:**
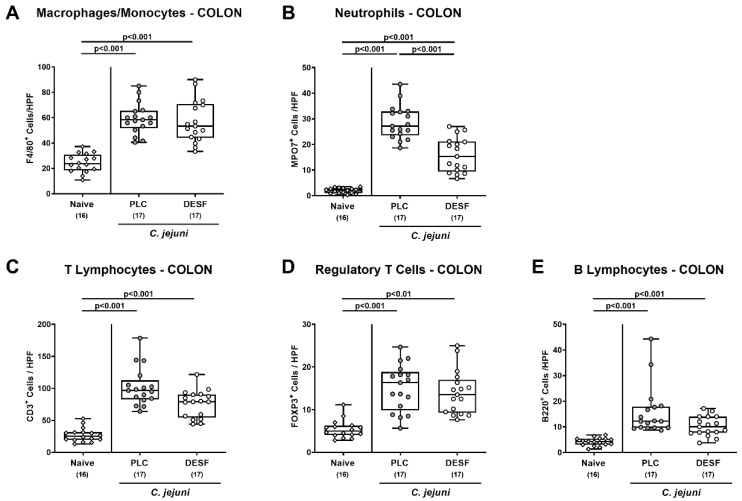
Immune cell responses in the colon following *C. jejuni* infection of microbiota-depleted IL-10^−/−^ mice that had been pretreated with deferoxamine. Microbiota-depleted IL-10^−/−^ mice were subjected to desferoxamine (DESF, white circles) pretreatment via the drinking water starting 7 days prior infection or received autoclaved tap water only (PLC; grey circles). On days 0 and 1, mice were perorally infected with *C. jejuni* strain 81-176. On day 6 post-infection, distinct immune cell populations were assessed in colonic paraffin sections by quantification of (**A**) F4/80^+^ macrophages and monocytes, (**B**) MPO7^+^ neutrophils, (**C**) CD3^+^ T lymphocytes, (**D**) FOXP3^+^ regulatory T cells, and (**E**) B220^+^ B lymphocytes within the colonic mucosa and lamina propria following immunohistochemical staining with respective antibodies. Indicated are the medians of positively stained cells within six representative high-power fields (HPF, 400 × magnification). Uninfected and untreated mice served as negative controls (white rhombus). Whiskers indicating the total range, boxes indicating the 25th and 75th percentiles of the medians (black bars inside boxes), significance levels (*p* values) calculated by the ANOVA test and Tukey’s post-correction (**A**,**B**,**D**) and the Kruskal–Wallis test with Dunn’s post-correction (**C**,**E**), and the total numbers of analyzed mice (in parentheses) are given. Shown data were pooled from three independent experimental sets.

We then addressed potential immune-modulatory properties of DESF and tested whether respective pretreatment was associated with changes in the abundance of innate and adaptive immune cell populations in the infected large intestines. Therefore, we again performed in situ immunohistochemical analyses, stained colonic paraffin sections with distinct antibodies and enumerated positively stained cells in the colonic mucosa and lamina propria. On day 6 p.i., mice from both cohorts displayed markedly increased numbers of innate immune cell populations such as F4/80^+^ macrophages and monocytes as well as of MPO7+ neutrophilic granulocytes in their colonic mucosa and lamina propria (*p* < 0.001 versus naive; Figure 4A,B), which also held true for adaptive immune cell subsets including CD3^+^ T lymphocytes, FOXP3^+^ regulatory T cells and B220^+^ B lymphocytes (*p* < 0.01–0.001; Figure 4C–E). Following DESF pretreatment, however, *C. jejuni* infected mice exhibited lower numbers of neutrophilic granulocytes in their colon when compared to PLC controls (*p* < 0.01; Figure 4B), whereas a trend towards lower large intestinal T cell counts were observed in the former versus the latter (*p* > 0.05, n.s.; Figure 4C). Hence, DESF pretreatment resulted in less *C. jejuni* induced colonic accumulation of neutrophilic granulocytes.

### 3.5. Intestinal IFN-γ Secretion Following C. jejuni Infection of Microbiota-Depleted IL-10^−/−^ Mice That Had Been Pretreated with Deferoxamine

**Figure 5:**
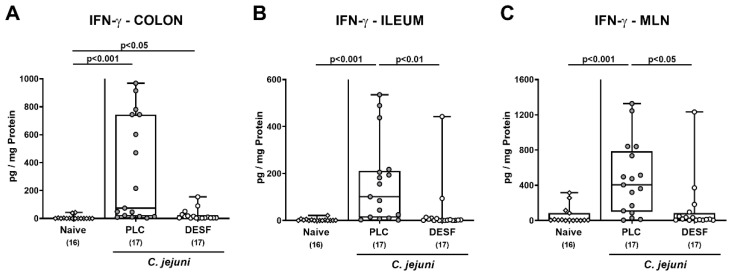
Pro-inflammatory IFN-γ responses in distinct parts of the intestinal tract following *C. jejuni* infection of microbiota-depleted IL-10^−/−^ mice that had been pretreated with deferoxamine. Microbiota-depleted IL-10^−/−^ mice were subjected to desferoxamine (DESF, white circles) pretreatment via the drinking water starting 7 days prior infection or received autoclaved tap water only (PLC; grey circles). On days 0 and 1, mice were perorally infected with *C. jejuni* strain 81-176. On day 6 post-infection, IFN-γ concentrations were measured in supernatants taken from overnight cultures of intestinal ex vivo biopsies such as the (**A**) colon, (**B**) ileum, and (**C**) mesenteric lymph nodes (MLN). Uninfected and untreated mice served as negative controls (white rhombus). Whiskers indicating the total range, boxes indicating the 25th and 75th percentiles of the medians (black bars inside boxes), significance levels (*p* values) calculated by the Kruskal–Wallis test with Dunn’s post-correction, and the total numbers of analyzed mice (in parentheses) are given. Shown data were pooled from three independent experimental sets.

We further measured pro-inflammatory IFN-γ secretion in ex vivo biopsies derived from distinct parts of the intestinal tract. On day 6 p.i., increased IFN-γ concentrations were measured in the colon from both PLC and DESF treated mice (*p* < 0.001 and *p* < 0.05 versus naive, respectively), whereas a trend towards lower levels in the latter versus the former could be observed (*p* > 0.05, n.s.; Figure 5A). In the ileum and in the MLN, however, enhanced *C. jejuni* induced IFN-γ secretion were observed in PLC as opposed of DESF pretreated mice (*p* < 0.001 versus naive; *p* < 0.01 and *p* < 0.05 versus DESF, respectively Figure 5B,C). Hence, DESF pretreatment dampened IFN-γ secretion in the intestinal tract of *C. jejuni* infected mice to basal levels.

### 3.6. Pro-Inflammatory Mediator Responses in Extra-Intestinal Compartments Following C. jejuni Infection of Microbiota-Depleted IL-10^−/−^ Mice That Had Been Pretreated with Deferoxamine

**Figure 6:**
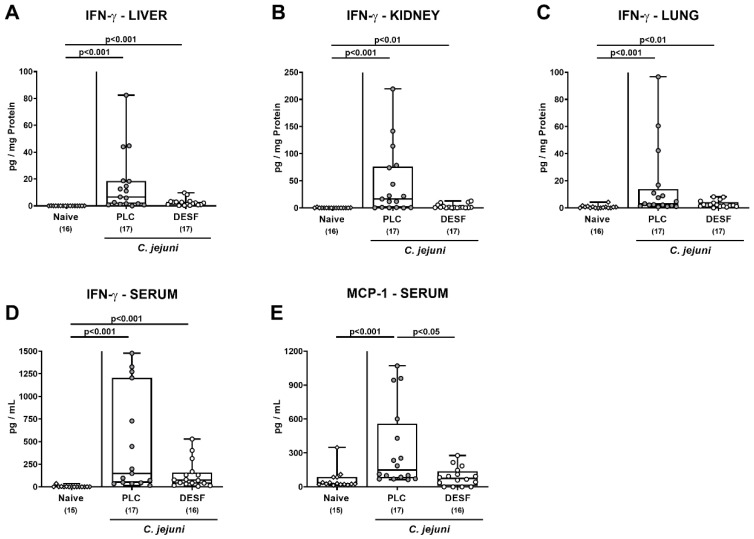
Pro-inflammatory mediator responses following *C. jejuni* infection of microbiota-depleted IL-10^−/−^ mice that had been pretreated with deferoxamine. Microbiota-depleted IL-10^−/−^ mice were subjected to desferoxamine (DESF, white circles) pretreatment via the drinking water starting 7 days prior infection or received autoclaved tap water only (PLC; grey circles). On days 0 and 1, mice were perorally infected with *C. jejuni* strain 81-176. On day 6 post-infection, IFN-γ concentrations were measured in supernatants taken from overnight cultures of extra-intestinal ex vivo biopsies such as the (**A**) liver, (**B**) kidney, and (**C**) lung and in (**D**) serum samples. In addition, (**E**) MCP-1 secretion was assessed in serum samples. Uninfected and untreated mice served as negative controls (white rhombus). Whiskers indicating the total range, boxes indicating the 25th and 75th percentiles of the medians (black bars inside boxes), significance levels (*p* values) calculated by the Kruskal–Wallis test with Dunn’s post-correction, and the total numbers of analyzed mice (in parentheses) are given. Shown data were pooled from three independent experimental sets.

We further tested if the immune-modulatory effects of exogenous DESF were restricted to the intestinal tract or also affected extra-intestinal sites including systemic compartments of *C. jejuni* infected mice. On day 6 p.i., comparably increased IFN-γ concentrations were measured in the liver, kidney, and lung ex vivo biopsies as well as in serum samples taken from mice of both cohorts (*p* < 0.01–0.001; Figure 6A–D). In sera from PLC, but not DESF pretreated mice, however, enhanced *C. jejuni* induced MCP-1 secretion were observed (*p* < 0.01 versus naive; *p* < 0.05 versus DESF; Figure 6E). Hence, DESF pretreatment of *C. jejuni* infected mice was associated with dampened systemic MCP-1 secretion.

## 4. Discussion

The key results of this pilot study indicate for the first time that iron chelation by DESF exerts disease-ameliorating effects in a murine campylobacteriosis model. Unexpectedly, DESF application did not exert antimicrobial effects against *C. jejuni* in the gastrointestinal tract of mice even though DESF was applied in a very high concentration of 500 mg/L and hence, fifty times higher than the minimal inhibitory concentration (MIC) of DESF against *C. jejuni* reported earlier [40]. This was confirmed independently by us given a MIC of DESF as low as 8 mg/L tested against the *C. jejuni* strain 81-176 (data not shown). Thus, given the 1:1 molar binding ratio of DESF::iron, the here applied DESF concentration should have been sufficient to exert antimicrobial effects in vivo. This assumption is further supported by the iron concentration of ≈160 mg/kg in the mice feeds which was not sufficient to overcome the iron depletion established by oral DESF at the applied concentration. In this context, the finding that gastrointestinal pathogen loads were not reduced in DESF treated mice is remarkable and it is tempting to speculate that iron bound to the host molecules lactoferrin, transferrin or heme, which is not accessible to chelation by DESF might fuel *C. jejuni* growth [33]. This offers an attractive perspective for the application of DESF treated, microbiota-depleted mice to study roles of host-derived iron molecules in the intestinal colonization capacity of *C. jejuni*. These future investigations using *C. jejuni* mutants deficient in host iron uptake systems could be completed by the investigation of the effect of iron deprivation on the virulence of *C. jejuni*. Since iron is a key signal molecule for environmental sensing of *C. jejuni*, pathogenicity could be substantially reduced by the DESF mediated inhibition of motility, which might indeed explain the amelioration of symptoms despite effective gastrointestinal colonization at high loads. In this context, it is important to note that non-motile mutants of *C. jejuni* did effectively colonize microbiota-depleted IL-10^−/−^ mice without induction of any symptoms [50].

On the other hand, the improved clinical outcome in *C. jejuni* infected mice that had been pretreated with DESF points towards a significant effect of the drug in ameliorating campylobacteriosis. Microscopic analysis of the colon tissues revealed that DESF treatment mainly protects the epithelial barrier by inhibition of apoptosis, which in turn dampens intestinal and systemic inflammation in the course of *C. jejuni* induced enterocolitis. The inhibition of both apoptosis and inflammation by DESF is supported by the fact that iron is a strong inducer of apoptosis [51] and is further well in line with earlier studies showing that DESF treatment ameliorated iron-induced intestinal apoptosis and inflammation in rats [52]. Taken together, these findings support the view that iron deprivation by DESF protects *C. jejuni* infected mice from epithelial cell death, which in turn results in reduced intestinal inflammation and in the better clinical outcome of disease observed in treated animals. In addition, DESF can protect the intestinal tissues from iron-induced ferroptosis, constituting a second form of non-apoptotic epithelial cell death triggering barrier disruption and aggravation of intestinal inflammation, as shown recently for ulcerative colitis patients [53].

The shown reduction in pathogen-induced IFN-γ concentrations in the MLN draining the infected intestines, and in the ileum derived from DESF-challenged mice point towards direct anti-inflammatory effects of iron chelation in campylobacteriosis and hence, expands our knowledge on the potent immune-modulatory functions of iron in inflammation. Several previous studies could show that iron chelation by DESF influences intestinal inflammation directly, leading to changes in key mediator concentrations including interferons and interleukins [54].

Remarkably, DESF pretreatment of mice resulted in a significant reduction in *C. jejuni* induced systemic MCP-1 concentrations to basal levels. This DESF-mediated protection of *C. jejuni* infected mice from systemic immune responses is well in line with the earlier findings that iron chelation exerts inhibition of systemic inflammation in endotoxin shock [55,56] and in atherosclerosis [57], which was further substantiated by downregulation of inflammation in aortic endothelial cells [58].

## 5. Conclusions

The presented promising and, in part, unexpected findings highlight the applied murine campylobacteriosis model as a reliable tool for further analysis of the role of bacterial iron uptake and metabolism as well as of iron chelation by DESF in campylobacteriosis. The underlying molecular mechanisms by which DESF exerts its disease-alleviating effects are unclear and exceed the focus of this preclinical placebo-controlled intervention study. However, the obtained results may support a role of host iron sources in intestinal colonization of *C. jejuni* and of iron per se in the onset and course of campylobacteriosis, which need to be investigated in more detail in future studies.

## Data Availability

The corresponding author provides the data from this study on request.
